# Are there reasons behind high Handrub consumption? A French National in-depth qualitative assessment

**DOI:** 10.1186/s13756-022-01074-2

**Published:** 2022-02-23

**Authors:** Delphine Berthod, Dara Alvarez, Anne Perozziello, Fanny Chabrol, Jean-Christophe Lucet

**Affiliations:** 1 University of Paris, INSERM IAME, U1137, Team DeSCID, Paris, France; 2 Infectious Diseases Department, Central Institute of Valais Hospitals, Sion, Switzerland; 3 AP-HP, Infection Control Unit, Bichat-Claude Bernard University Hospital, Paris Cedex 18, France; 4 Université de Paris, Institut de Recherche pour le Développement (IRD), INSERM, Centre Population et Développement (Ceped), Paris, France

**Keywords:** Cross infection, Prevention and control, Hand disinfection, Standards, Health personnel, Education, Health knowledge, attitude practice, Qualitative research, France

## Abstract

**Background:**

Hand hygiene (HH) is the most important measure for preventing healthcare-associated infections. A significant correlation between alcohol-based handrub consumption (AHRC) and observed HH compliance rates has been established. In France, publicly reported AHRC displayed a large heterogeneity across healthcare facilities (HCFs). We aimed to describe programmes for promoting HH in the top and medium AHRC scorers and to assess factors and drivers leading to a high AHRC score in a panel of French HCFs.

**Methods:**

We performed a nationwide qualitative comparative case study based on in-depth semi-structured interviews in 16 HCFs with high, 4-year AHRC scores, and a sample of seven university hospitals (UHs) with medium AHRC scores. Infection Prevention and Control Team (IPC) members (n = 62), quality managers/chief executive officers (n = 23) and frontline workers (n = 6) were interviewed, using a grounded theory approach and an iterative thematic approach.

**Results:**

Ninety-one interviews were performed. There was a large heterogeneity in IPC structures and objectives, with specific patterns associated with high AHRC that were more organisational than technical. Four areas emerged: (1) strong cohesive team structure with supportive and outcome-oriented work attitude, (2) IPC structure within the organization, (3) active support from the institution, (4) leadership and role model. Among high AHRC scorers, a good core IPC organisation, a proactive and flexible management, a frequent presence in the clinical wards, and working in a constructive safety climate were prominent.

**Conclusion:**

We highlighted that IPC structure and activity is heterogeneous, with organisational and behavioural characteristics associated with high AHRC score. Beyond technical challenge, our work underlines the importance of strong structure of the IPC and behavioural approaches in implementing key IPC programmes.

## Background

Hand hygiene (HH) is the most important prevention measure of healthcare-associated infections (HAIs) and transmission of multidrug-resistant organisms (MDRO) [1]. Significant correlation between alcohol**-**based handrub consumption (AHRC) and HH compliance rates [2, 3], and between AHRC and MDRO reduction has been established [4]. In France, publicly reported AHRC scores displayed a large heterogeneity across healthcare facilities (HCFs) [5]. In Germany, quantitative crude data of the distribution of AHRC are provided for benchmarking between hospitals in the German national surveillance system (KISS) [6].

Studies suggest that a behavioural approach, based on education, audits and performance feedback and resolution of local challenges, could be as important as the recommended precautions themselves [7, 8]. Of the individual factors influencing HH compliance, barriers have been identified, including product (skin irritation…), personal/behavioural (risk perception, motivation) and organisational (lack of available alcohol-based handrub (AHR) or lack of time) issues, all 3 being strongly intertwined [9]. Several studies have focused on institutional factors that can influence HH compliance at the level of the clinical wards [10, 11], or local institution [12–14], but few considered these issues from the Infection Prevention and Control (IPC) teams viewpoint.

A socio-anthropological approach can help to step beyond the efficacy analysis of HH promotion programmes, with a view to assessing programmes in their local and organisational context, at the scale of clinical wards and institutions. Here we aimed to assess factors and mechanisms leading to a high AHRC score in a panel of French HCFs and compare IPC organisation between top and medium scorers.

## Methods

### Study design

We conducted a qualitative comparative case study based on in-depth semi-structured interviews. Our study followed the Standards for Reporting Qualitative Research recommendations [15].

The AHRC index has been described elsewhere [5]. Briefly, 12 categories of HCF across the 2546 (data from 2016) HCFs in France were identified. The AHRC index is computed by the ratio of actual to predetermined expected HH procedures per day and per patient category, e.g. from medical and surgical wards, intensive care units or emergency department. Based on a volume of 3 mL per HH and the number of patient-days in each unit category, a yearly AHRC is computed and benchmarked against other HCFs in each HCF category.

Inclusion criteria were HCF categories accommodating beds of acute care and rehabilitation with the highest AHRC scores. HCFs with only paediatric beds, haemodialysis, one-day care, psychiatric care, long-term care and nursing homes were excluded, leading to 2019 preselected HCFs. Sixty HCFs had AHRC scores higher than the 90th percentile of the minimal expected AHRC within their HCF category from the national 2013–2016 database provided by the French National Authority for Health (HAS) (Table 1).

**Table 1:** French healthcare facilities with high AHRC scores

Healthcare facility	Number of HCFs in each category	Number of HCFs with AHRC higher than the 90th percentile (%)
University hospital	71	18 (25)
General hospital > 500 beds	204	5 (2)
General hospital < 500 beds, private clinic	803	16 (2)
Local hospital/rehabilitation centre	924	14 (2)
Regional cancer centre	17	7 (41)
Total	2019	60 (3)

Among the 60 eligible HCFs, a convenient sample of 16 was selected, based on their higher AHRC scores and the following criteria:

- Four among the 71 university hospitals (UHs), the two highest scorers of the Greater Paris University Hospital network except one that had been visited during the pilot phase (described later) and two in other French regions;
- Four among the 204 large general hospitals (GHs): 3 in Northern France and one in the Paris area; the last one with high AHRC score had been visited during the pilot phase;
- Eight others HCFs were the higher scorers of their category and purposively selected as being likely to give a broad perspective of response patterns: five small hospitals or rehabilitation centres that were distinct in size and structure, and three geographically distant regional cancer centres.

In a second study phase, we compared the results from the four UH top scorers with a convenient sample of seven UHs with medium AHRC scores (scoring between 75 and 95%, see Table 4).

### Data collection

Between September 2018 and March 2019, face-to-face semi-structured interviews were conducted by one female social science researcher (DA) with the help of a semi-structured interview guide. DA was supervised by an experienced qualitative researcher (AP) in designing the interview guide and performing test interviews.

The interview guide consisted of open-ended questions to explore participants’ views on and experiences with HH and IPC functioning in general. These open-ended questions were built within defined areas of interest following a grounded theory approach. The interview guide was tested for extensiveness in the primary investigator’s UH, in one another Paris high-scoring UH and one large high-scoring GH. The targeted interviewees were a convenient sample composed of the members of the IPC team, representatives of the HCF administration, medical management, members of the quality management team and head nurses or link nurses.

### Analysis

All interviews were audio recorded, de-identified and transcribed verbatim. Interviews were analysed independently by DA and by an infectious diseases specialist with experience in infection and prevention (DB) using an iterative thematic approach [16]. This resulted in the definition of themes and subthemes confirmed or re-evaluated by ongoing data analysis. An analytical framework was used to compare interviews. Themes and subthemes were then discussed and validated with AP and JCL.

Ethics approval was obtained from the Institutional Review Board (CEERB Paris Nord 00006477) and all participants signed informed consent prior to interview. We received an unrestricted grant from Anios Company, which had no role in the study design, data collection, data analysis, data interpretation or writing of the manuscript.

## Results

### Participants

**Fig. 1:**
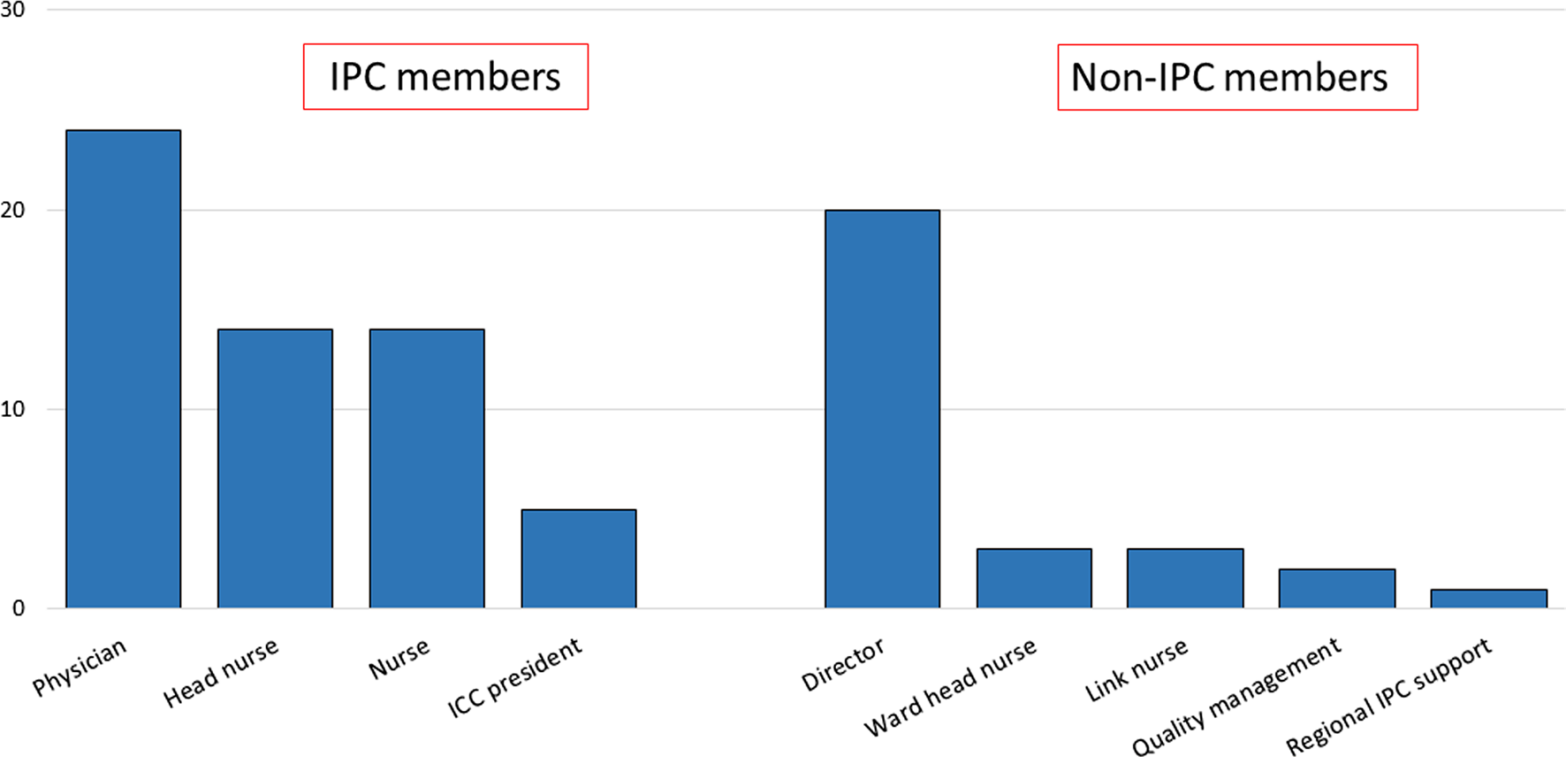
Profile of interviewees

Participants’ hospitals and interviewees’ details are summarised in Table 4. Overall, 91 interviews (about 64 h) were conducted, with an average of 3.4 (range 1–8) interviews per site, depending on the size of the HCF; 68% (62/91) of the interviewees were IPC members (Fig. 1).

There was a large heterogeneity in IPC structures and programmes with specific patterns associated with high AHRC. Recurrence of overarching themes was reached quickly regarding technical issues like provision and availability of handrub products, with no emerging differences between top and medium scorers.

Four areas shown in Table 2 emerged as facilitators for HH compliance: (1) strong cohesive team structure with supportive and outcome-oriented work attitude; (2) IPC structure within the organisation of the healthcare facility; (3) active support from the institution; (4) leadership and role model. Illustrative participants’ quotations of top (+) and medium (−) scoring HCFs are presented in the text for the main ones and in Table 3.

**Table 2:** Panel–Major themes and components emerging from interviews

**Strong cohesive team structure with supportive and outcome-oriented work attitude** *Human resources* *Team spirit/internal communication*
**IPC structure within the organisation of the healthcare facility** *Location of the IPC team and presence in the frontline workplace* *Timeliness of attitude towards clinical events—reactive or proactive* *Flexible and positive communication* *Culture of auditing and feedback* *AHR consumption indicator (ICSHA)*
**Active support from the institution**
**Leadership and role model**

**Table 3:** –Verbatim transcriptions of audiotaped comments by health professionals – except verbatim transcription already cited in the text

	Observation	Verbatim transcripts
Strong cohesive team structure with supportive and outcome-oriented work attitude	Human resources	
	Team spirit / internal communication	*"We really have to be a team, so we really have to get across the same messages" IPC MD and nurse UH3(* +*)* ^a^
	Recruitment: specific choice or attractiveness for prevention control and interdisciplinary job	*"Because I know at least two nurses, one of whom was in the unit, who are at home with a long illness and could come and work on those projects.… There's a lot of absenteeism like everywhere in French hospitals."* *IPC MD ICC president UH7(−)*
	Inter-hospital networking	*"The overall scores of all the national indicators are higher in the cancer centres. There's the whole history because oncology (…), it's always seeking innovation." IPC MD CC3 (* +*)* ^b^
IPC structure within the organisation of the healthcare facility	Visibility of the IPC team	*"We absolutely wanted the infection control team to be at the centre of the care facility." IPC MD and nurse UH3(* +*)* ^c^
	Presence in the frontline workplace	*"It's only during an epidemic that we don’t demand appointments." IPC MD UH11(−)* ^d^ *"We try not to intrude in the healthcare units." IPC head nurse UH11(−)* *"We can't be onsite outside a crisis situation. So, they only see us during a crisis." IPC MD UH6 (−)*
	Timeliness of attitude towards clinical events—reactive or proactive	*"That gives us so many things (to think about) afterwards. We can see, if we've acted on a subject, we'll see in the following months…so we can see if it's simmering, if it's increasing or not." IPC head nurse UH1(* +*)*
	Defining priorities—opportunity costs	*"I think team mobilization for the management of patients with emergent highly-resistant bacteria, that's diverted our energy somewhat" IPC MD UH10(−)*
	Flexibility in communication; positive communication	*"Whenever a nurse turns up wearing jewellery, it's like no, you're not working if you have jewellery." IPC nurse RC5(* +*)* ^e^ Positive communication *"Being tactful: if we oppose a team or someone, forget it. Afterwards, it's very hard to backtrack." IPC nurse CC3(* +*)* *"Teaching can't just be made up." IPC nurse UH6(−)* *"We, we have communication problems. (….) We lay out guidelines. Someone said to me regarding staff hierarchy, all the experts talk about guidelines (…). It's too light a term. And obligation is too bossy. So, it's odd." IPC nurse UH11(−)* *"When some people, certain doctors, when they're working a huge amount, …you can't say anything to them, because they send you packing." IPC MD UH7(−)* Cynicism and discouragement *"Even if from time to time we have to swallow one insult after another, it's our job." IPC MD UH8(−)*
	Culture of auditing and feedback	*"When a carrier of multi-resistant bacteria arrives, the whole department is informed and we conduct three audits a week, before reporting straight after." IPC head nurse UH4 (* +*)* *"And we had a 'zero jewellery' audit twice a year, so we went into all the departments. (…) And then, we took the opportunity to talk about hand sanitizer. So, there was still the feedback and the posters for the personalized service for the department. That's our thing, each time we send the personalized results by department and even, at the same time, the hand-rub consumption (….)." IPC MD CC3 (* +*)*
	Frontline workers staffing in a difficult sociopolitical context	*"There are never the same people at meetings. They're always representatives of representatives, so we have to explain all over again each time. It's pointless meetingitis." IPC head nurse UH4 (* +*)*
	Link nurses	*"We had correspondents. This network collapsed (…) over time people changed sector and were not replaced." IPC MD UH5(−)* *"It's true the role of link nurses is essential because in fact they're peers. They're on an equal footing and so can discuss more freely. When there's a hierarchical relationship, it's much more complicated." Manager, care RC3 (* +*)*
	AHR consumption indicator (AH RC)	*"Because once we improve, the hand-rub consumption indicator changes and, hey, we're bad again!" ICC president MD UH5(−)* There were big differences also in the feedback of alcohol-based handrub consumption to the wards *"If we send the hand-rub consumption indicator, we don't get much feedback." IPC nurse UH6(−)* *"This indicator, it's completely…well, I find it absurd, in fact. No account is taken of the reduction of hospital-acquired infections in the departments. It can't be used as an argument among professionals." IPC MD UH8(−)* *"Using this indicator alone to have an idea of the prevention of hospital-acquired infections in a care facility, I find that very simplistic." IPC MD UH6(−)* *"The fact that there's an indicator associated with hand-rub use raises visibility and importance." IPC MD CC2(* +*)* *"If your organisation is not up to scratch, you'll have nosocomial infections, which is why proof is needed of the efficiency of the organization put in place." Quality manager UH3(* +*)*
Active support of institution		*"There are tensions at the hospital. (…) we're asked to do more with less, whether it's funding or staff." IPC MD UH6(−)* *"There absolutely no culture of quality, of prevention, of risk management at the hospital." (−) IPC MD UH6(−)*
	Infection Control Committee	"*We'd really like our public health physicians to speak to the doctors, but when they do it's within the confines of the medical committee and they have 5 min to get everyone on board (….)." IPC nurse UH6(−)* ^f^
Leadership and role model		*"Knowing one's hospital, each other's problems, knowing how the procedures are, or are not, applied." IP MD UH1(*+*)* *"I think there's drive, and a good atmosphere too. I think a good atmosphere at work also encourages good practice. The pleasure of being at work, the pleasure of working well." IPC nurse RC5(*+*)*

(+) top scoring HCFs and (−) medium scoring HCFs

### Composition, team culture and work attitude of the IPC team

A trend towards understaffing of infection control practitioners in the medium-scoring UH IPC teams (median, 0.83/1000 beds) was observed as compared with high-scoring UH IPC teams (median, 1.29/1000 beds) (Table 4). There was no difference in the nurse staffing (2.35 and 2.33/1000 beds, respectively).

**Table 4:** - Description of Healthcare Facilities and Interviewees

	Beds (n)	IPC team/1000 beds (Practitioner/nurse)	Number of interviewees (IPC, non-IPC)	Average AHRC score 2013–2016 (%)
UH1 High scorer	2100	1.1 1.9	3 2	152
UH2 High scorer	700	1.4 3.6	1 0	141
UH3 High scorer	3000	0.7 2.8	3 2	164
UH4 High scorer	543	2.4 1.1	3 2	143
UH5 Medium scorer	2000	0.8 2.5	3 1	76
UH6 Medium scorer	2600	0.6 1.4	5 1	95
UH7 Medium scorer	1400	1.4 2.1	6 2	93
UH8 Medium scorer	1300	1.2 3.1	2 0	92
UH9 Medium scorer	1700	1.3 1.2	3 0	92
UH10 Medium scorer	2400	0.8 2.5	4 1	101
UH11 Medium scorer	3000	0.8 2.3	3 1	72
GH1 High scorer	1000	1.6 4.0	2 2	127
GH2 High scorer	600	1.3 4.2	4 0	133
GH3 High scorer	2000	0.75 1.7	2 2	130
GH4 High scorer	700	1.4 2.9	1 0	132
RC1 High scorer	42	11.9 35.7	2 2	203
RC2 High scorer	476	2.1 2.1	3 1	141
RC3 High scorer	163	0 6.1	2 4	116
RC4 High scorer	230	0.3 3.7	2 2	172
RC5 High scorer	25	0.8 2.8	1 3	269
CC1 High scorer	450	0.9 4.4	1 0	131
CC2 High scorer	220	4.6 4.6	2 1	134
CC3 High scorer	170	3.5 5.9	4 0	132

UH university hospital, GH general hospital, RC rehabilitation centre, CC cancer centre

IPC infection prevention and control, AHRC alcohol-based handrub consumption

Practitioner corresponds to medical doctor or specialized pharmacist

#### Team culture/internal communication

Good communication between IPC members was critical. In most high-scoring UHs, a weekly ~ 2-h meeting was organized where the objective was to agree upon a message for external communication (verbatim ^a^).

Lack of team cohesion was noted in some medium-scoring IPC teams: between IPC nurses either because of interpersonal difficulties (UH5(−)) or due to time constraints (UH7(−)); between infection control practitioner and nurses because of lack of interest from the IPC’s leader about “real-life” problems (UH9(−)) or prioritising of research activities (UH6(−)).

The IPC team was more effective if the workforce had specifically chosen to work in an IPC position and did not consider it as a second option after leaving frontline work. *“That's why I chose an interdisciplinary job. It's brilliant to go and see what the others are doing, I've always found that, even though we don't have in-depth knowledge of all the subjects.” IPC MD CC3(+)*.

In contrast, the medium scorers reported difficulty in recruiting IPC nurses:

*“Because we already have… for us, infection control nurses, it's practically only people who are… who are reclassified because they are no longer suited to technical activities.” IPC MD ICC president UH7(*−*).*

#### Inter-hospital networking

IPC teams in high-scoring HCFs often participated to an inter-hospital network where common objectives were shared and regular inter-hospital meetings were organised. For example, four out of the 5 French high-scoring GHs were located in northern France, close to a high-scoring UH. This was also observed in the Greater Paris University Hospital network (AP-HP), as well as between the regional cancer centres (verbatim ^b^).

In contrast, IPC teams from medium-scoring UHs might feel isolated and abandoned (UH6(−)).

### Organisational and structural aspects within the hospital and networks

Location of the IPC working office close to the clinical wards was perceived as critical for visibility and recognition in the HCF (verbatim ^c^).

A frequent presence of the IPC teams in the frontline workplace was reported to be critical in the high-scoring HCFs, with IPC members being seen as peers. *“There are few of us in our office. The clinical wards know us.” IPC MD UH1(+)*.

In contrast, in several medium-scoring UHs (UH6 (−), UH9(−), UH11(−)), IPC members went to the frontline workplace only if there was a request from the clinical unit or after having made an appointment with them (verbatim ^d^).

#### Timeliness of attitude towards clinical events—reactive or proactive

The reaction towards outbreaks or epidemiological events was distinct between high and medium scorers. The latter rather waited for problems to react. Thus, their presence in the frontline workplace was frequently perceived by the clinical staff with an ongoing problem.* “So, we're often putting out fires. And normally, the role of the supervisor is to go in first… before the fire, to put things in place, to try and organize it. It's much more complicated.” IPC head nurse UH7(−).*

In contrast, high-scoring hospitals reported a proactive policy through, for example, regular infection risk visits in all clinical wards.

#### Flexible and positive communication

A “skilled hygienist” was evidenced in small top-scoring HCFs, where the prominence of the IPC head nurse made recommendations easier to agree with (RC5 (+) verbatim ^e^).

Adapting messages to professional groups was deemed important: sharing scientific arguments with physicians, and more practical considerations with nurses. *“We're in the clinical staff with the results of scientific studies.” IPC MD UH3(+).*

Supportive comments towards actionable solutions rather than criticism. *“The infection control team comes into the department and it's never taken as an inspection, never as a criticism, it's really taken as improving practices. And it's important it's experienced like that, in fact.” non IPC head nurse UH4(+)*.

In the medium-scoring UHs, some interviewees admitted difficulties in communication, which they sometimes attributed to deficient communication skills. *“I see them. They leave the department and don't use hand-rub (…) What can be done? Challenge them? Perhaps what's needed is to be the watchdog that barks at them. Sometimes it's very difficult.” IPC nurse UH5(−)*.

#### Culture of auditing and feedback

Audits of compliance with HH were regularly performed in all HCFs, sometimes with adaptive protocols in high-scoring HCFs. *“It's a bit harder to reach the night shift, so the infection control team suggested trying self-audits. Everyone can audit someone else, regardless of rank, role, position.” Manager, care UH4(+)*.

In contrast, poorly staffed IPC teams reported a lack of human workforce as responsible for deficient feedback after auditing. *“Audit feedback, on the other hand, is extremely complicated because we can no longer mobilize the teams. (…) But we must free up the professionals and, well, for the latest audits…We don't manage to provide feedback.” IPC MD UH5(−)*.

#### AHR consumption indicator (ICSHA)

The 4-year average ICSHA score was 153% in the 16 top scorers and 89% in the 7 UH medium scorers. We noticed a large heterogeneity in the use of the AHRC index. Many medium-scoring UHs did not relate the AHRC index to the actual situation in their hospital, and some were not aware of the AHRC score in their HCF, including in the quality and risk management department. *“Then, I don't know how we're classified, if we're good, or not. It seems to me we're not bad. They'll say we're within the norms.” Quality manager UH7(−)*.

In medium-scoring UHs, there were gaps in displaying AHRC score in the clinical wards. *“Me, I send the handrub consumption indicator and then tell them ‘get back to us if you want a more in-depth presentation of the results and we'll discuss them.’” IPC MD UH5(−).*

In the top-scoring hospitals, they considered AHRC rather as a leverage to communicate with frontline workers. *“The indicators are a marker, a benchmark, and that showcases the work. It guides them in their work.” IPC head nurse RC4(*+*).*

### Active support by the institution

A lack of support from the institution was often mentioned in the medium-scoring hospitals, with priority given to financial issues rather than quality of care. *“We're...we're in a department where, in fact, I mean, only the services that bring in money are important.” ICC president MD UH6(−).*

On the other hand, top-scoring hospitals felt supported by their management. *“And then the management, it's a genuine institutional policy. The management has always paid attention to us.” IPC head nurse RC4(+).*

In the medium-scoring UHs, the Infection Control Committee (ICC) was often not actively supported by the administration. For instance, a resigning ICC president was not replaced in one medium-scoring UH (verbatim ^f^).

### Leadership, champions and role model

Leadership was acknowledged among the top scorers to be very important. Multiple examples of key personalities within the top-scoring hospital IPC teams were found. In the rehabilitation and long-term centres their value was key.* (talking about the IPC head nurse) “She has the key, and her door is open. She's in all the analyses. I've got a little marvel there.” Manager RC2(+). “We all know each other. So, communication is easy. It's simpler to convey information, get messages across. To raise awareness among the professionals.” IPC head nurse RC4(+).*

Role modelling was very important especially from the head nurses in the wards. Boundary spanners could emerge in any role, for instance in link nurses. *(medical doctor talking about the head nurse of the ward) “Another thing we've also seen, which I think is very important, is the department's head nurse. If involved, the head nurse injects drive, leadership.” IPC MD UH3(+).*

## Discussion

Our national qualitative multicentre study revealed that strong core IPC organisation, proactive and flexible management of field activities, frequent presence in the clinical wards and an ability to adapt to local contexts, participation in an IPC inter-hospital network and working in a constructive safety climate with support from the institution and role modelling were prominent in the high AHRC scorers.

Previous studies have assessed the key factors for good adherence to HH [17], including qualitative studies [18, 19]. However, they mainly focused on frontline healthcare workers and their individual barriers and facilitators, while our scope was the IPC team and local organisation. Hospital organisation, management, and structure for prevention of HAIs has been extensively reviewed [20] at the institutional level. Other studies assessed the impact of hospital managers [12]. To our knowledge, ours is the first qualitative study to explore IPC functioning and key actors’ experiences with and points of view about HH. It is striking that the factors identified in our study were more of an organisational than technical or individual nature. For example, the choice of the AHR solution or the technique of the hand-rubbing did not appear to play a discriminant role in our analysis.

We selected the apparently most efficient IPC teams based on a high AHRC score. Although the usefulness of this score in reflecting the actual HH compliance remains debated, and although a correlation between high AHR consumption as a surrogate for quality and realities from IPC staff has not been qualitatively assessed yet, recent studies including many clinical wards over several years showed a correlation between AHRC score and compliance with HH [3]. The 60 HCF selected in our study were identified from all 2500 + French HCF over 4 years, which likely is independent from short-term variation in consumption, and broadly reflects the commitment of the HCF to improving HH.

Our study showed a large heterogeneity of AHR consumption in HCFs. UHs and reference cancer centres accounted for 4% of the 2019 French HCFs, but for 25% and 41%, respectively, of top scorers in their category, higher than the 90th percentile of the expected AHRC for their activity (Table 1). Belonging to a network could partly explain these high proportions, including the national network of reference cancer centres, with a long history of shared protocols and practices, and a high quality and safety culture. Ten out of the 18 AHRC top scorers from UHs were from the Greater Paris University Hospital network, AP-HP. Lastly, four out of the five top scorers in the group of large GHs were located in Northern France, surrounding a top-scoring UH, and having shared the management of several regional outbreaks [21, 22]. These networks were informal and not built on HAI surveillance, but rather on shared vision, practices and experience, as already observed [23, 24].

Beyond the high AHRC score, top scorers frequently used the unit-level AHRC score to prompt benchmarking and competition between units and departments and between HCFs. It was suggested that friendly competition helps increase HH compliance during a multimodal intervention programme [25]. In our study, top scorers used the indicator as an influential tool to communicate to lower users and propose local solutions. By contrast, medium UH scorers frequently viewed the AHRC score as biased, not related to reality, and imposing tedious institutional paperwork.

Comparing our results with the 10 crucial elements for the organisation of effective infection-prevention programmes in hospitals [20], the following ones were predominant in our top scorers’ findings: (1) working in a positive organisational culture that fosters working relationships and communication across units; (2) taking into account local conditions and identifying champions in the promotion of intervention strategies and (3) participating in a network.

As evidenced from qualitative studies [11, 26], it is critical to describe the interplay between context and interventions to understand the observed outcomes. Our study showed a large heterogeneity in IPC functioning. For example, in a top-scoring rehabilitation centre, the IPC head nurse served as a champion, had innovative ideas, was very well known and liked by her colleagues; her messages were easily applied. Moreover, positive deviance was important, with many nurses serving as link nurses helping to make HH the responsibility of everybody [11]. Champions and leaders in the IPC were deemed critical in implementing new strategies and supporting the requests, recommendations and actions of the IPC team toward administration and frontline workers [13, 27]. In contrast, some medium-scoring UHs suffered from lack of communication and cohesive team structure, sometimes fostered by a high turnover in the IPC teams. This could be overcome by presence in the field and proactive management regarding IPC emerging issues.

Safety culture can differ even in wards of the same hospitals [11]. We noticed in the top scorers a high capacity to adapt by delivering different messages to the specific and often less compliant category of doctors, for instance with evidence-based scientific articles instead of injunctions. According to Hofstede’s model of cultural dimensions, France has a high degree of power distance, indicating a high level of hierarchy and an unequally distributed power, as well as a high level of individualism [28, 29]. Restricting clinicians in their intentions and actions could be considered a hindrance to their work, as recently shown for antimicrobial stewardship [30, 31].

Two other key components emerged from the high-scoring HCFs. One was the presence of the IPC team in the hospital field, with adaptability and ability to answer concerns and questions in a proactive perspective. The second was proactive management of the IPC, in contrast to reactive ones in the medium scorers. Being proactive enhanced efficacy and improved the image of IPC teams as helpers instead of being “problem-associated”. This is congruent with the literature [32]. In a tertiary-care hospital in the Netherlands, Caris et al. found in a hospital-wide programme to enhance HH compliance that units with proactive or generative safety culture showed improvement or had high baseline compliance, while units with lower levels of safety culture did not [11]. These differences were striking because all units had enjoyed the same opportunities, resources, and support to improve compliance.

Finally, the safety climate in an institution was also felt to be important as previously reported [9], where the safety climate was deemed lower in medium- than in high-scoring UHs.

Our study has strengths and limitations. Strengths were its multicentre design involving a set of varied HCFs that were selected on the basis of actual consumption of AHR over four years, globally reflecting compliance with HH. Furthermore, all interviews were performed by a single researcher, with a multidisciplinary analysis. Regarding limitations, we cannot affirm that a high AHRC score was linked to high standard of IPC in the selected HCFs. Interviewees and the interviewer were not blinded to their AHRC score, which could have influenced the interpretation (on both sides) of their IPC functioning. Finally, 16 high-scoring HCFs of various sizes and activities were investigated, as compared to seven medium-scoring UHs only, which may be less representative of all HCFs. However, the selected panel of HCF and interviewees represented a variety of situations, programmes and perceptions.

## Conclusion

This qualitative study highlights that IPC structure and activity is heterogeneous, with organisational and behavioural characteristics associated with high AHRC score. Our work highlights the importance of a strong cohesive structure of the IPC team and of a behavioural approach in implementing key IPC programmes. Focusing on the frequent presence of IPC members in the wards and promoting a specific proactive management attitude seem to be implementable actions. Our findings offer an opportunity to improve IPC team functioning by implementing several operational strategies.

## Data Availability

Data will not be shared due to privacy and translation reasons.

## Abbreviations

HH: Hand hygiene
AHR: Alcohol-based handrub
AHRC: Alcohol-based handrub consumption
HCF: Healthcare facility
UH: University hospital
IPC: Infection Prevention and Control
HAI: Healthcare-associated infections
MDRO: Multidrug-resistant organisms
HAS: French National Authority for Health
GH: General hospital
CC: Cancer center
RC: Rehabilitation Center
ICC: Infection Control Committee
AP-HP: Greater Paris University Hospital Network
ICSHA: Index de consommation de solution hydro-alcoolique
